# The Essential Similarity of Innocent and Malignant Tumours

**Published:** 1907-09

**Authors:** 


**The Essential Similarity of Innocent and Malignant Tumours.** By Charles W. Cathcart, M.A., M.B. Bristol: John Wright and Co. 1907.

Some eighty-six admirably reproduced photographs are appended to seventy-nine pages of letterpress. The latter concerns itself with the question of the relations existent between innocent and malignant tumours. The writer sets out to prove that there is a definite demonstrable gradation from innocent to malignant tumours, that this transformation in character may sometimes be observed in the same tumours, and that combinations of character may sometimes be met with.

The examples of these conditions are chiefly drawn from bony growths, and while we must admit that a good case is made out, it is a moot point whether the conclusions drawn from bony growths may be applied to other tumours. However, the matter is one of general interest, and the surgeon will find a perusal of this book both suggestive and helpful.

The impression gained from the arguments advanced is an enforcement of the doctrine of "original sin." All tumours are deemed to possess latent malignancy, even though they appear to be temporarily innocent.

